# A Quantitative Serum Proteomic Analysis Helps to Explore the Comprehensive Mechanism and Identify Serum Biomarkers of Shengmai Injection’s Effect on Isoproterenol-Induced Myocardial Ischemia in Rats

**DOI:** 10.3389/fphar.2021.666429

**Published:** 2021-04-28

**Authors:** Xiaoping Zhang, Jie Zhang, Xiangyu Ji, Zhenzhen Wei, Baoyue Ding, Guoqiang Liu, Xiaoqing Lv, Yongxia Zheng, Shuyu Zhan

**Affiliations:** ^1^ Department of Science and Education, the First Hospital of Jiaxing, Affiliated Hospital of Jiaxing University, Jiaxing, China; ^2^ Department of Pharmacy, College of Medicine, Jiaxing University, Jiaxing, China

**Keywords:** Shengmai injection, serum proteomics, TMT, myocardial ischemia, serum biomarkers, glyceraldehyde-3-phosphate dehydrogenase, Fas apoptotic inhibitory molecule 3

## Abstract

Shengmai injection (SMI), a traditional Chinese medicine formula with the nature of multicomponent and multi-target, has been widely used in clinic for treating cardiovascular diseases in China; however, its comprehensive mechanism of action remains unclear. In this study, a TMT-based quantitative serum proteomics was performed to explore SMI’s global mechanism and help identify serum biomarkers of its effect on isoproterenol (ISO)-induced myocardial ischemia rats. The results of TMT-based proteomic analysis identified 227, 100, and 228 differentially expressed proteins (DEPs) for the model compared to the control group, SMI pretreatment + model compared to the model group, and SMI pretreatment + model compared to the control group, respectively. Based on bioinformatics analyses of gene ontology (GO), KEGG pathways, and the protein-protein interaction (PPI) networks for the DEPs, it is concluded that the comprehensive mechanism of SMI’s effect on ISO-induced myocardial ischemia injury includes regulation of energy metabolism, reducing endothelial cell permeability, regulation of vessel and cardiac contractility, anti-inflammation, and prevention of cell apoptosis. Furthermore, 10 common DEPs were found, and six of them were regulated in model vs. control group, while back-regulated in SMI pretreatment + model vs. model group. Among them, three functional proteins of glyceraldehyde-3-phosphate dehydrogenase (GAPDH), Fas apoptotic inhibitory molecule 3 (FAIM3), and uncharacterized protein (M0R5J4), which were verified by the PRM analysis, might be the potential serum biomarkers on SMI’s effects. Overall, this serum proteomics of SMI not only provides insights into the comprehensive mechanism underlying SMI’s effects on ischemic heart disease but also helps identify serum biomarkers for directing SMI’s cardioprotective effects.

## Introduction

Shengmai injection (SMI), a traditional Chinese medicine (TCM) formula composed of Panax ginseng C.A.Mey., Ophiopogon japonicus (Thunb.) Ker Gawl., and Schisandra chinensis (Turcz.) Baill., is widely used in clinic for treating cardiovascular diseases in China. According to the systematic reviews and meta-analysis, compared with Western medicine alone, SMI adjuvant therapy significantly improved cardiac function of patients with intradialytic hypotension (Chen et al., 2013) or chronic heart failure (Wang et al., 2020). And recent meta-analysis also showed SMI add-on therapy increased efficacy for chemotherapy in patients with non–small cell lung cancer (Duan et al., 2018) and the treatment of chronic obstructive pulmonary disease (Huang et al., 2019). SMI’s favorable clinical applications have attracted great interests in pharmacological studies, especially for its cardioprotective effects. It showed SMI protected cardiomyopathy by alleviating myocardial endoplasmic reticulum stress and caspase-12–dependent apoptosis (Chen et al., 2015a), or inducing myocardial mitochondrial autophagy *via* caspase-3/beclin-1 axis (Cao et al., 2020). SMI prevented myocardial ischemia reperfusion injury by reducing myocardial apoptosis and enhancing vascular endothelial growth factor (VEGF)–related angiogenesis (Lin et al., 2018). In addition, the related mechanism studies revealed SMI alleviated oxidative stress by activation of AKT and inhibition of ERK pathways (Zhu et al., 2019) and suppressed cardiomyocyte hypertrophy *via* activation of the AMPK signaling pathway (Li et al., 2019). However, all these studies focusing on SMI’s minority action targets and its comprehensive regulation mechanisms with the nature of multicomponent and multi-target are still poorly understood. Moreover, it is still lacking reasonable and accessible biomarkers for SMI’s treatment in cardiovascular diseases.

Systems biology methodologies, such as genomics, proteomics, and metabolomics, have showed significant advantages in studying the specific symptoms in TCM and the herbal formula’s comprehensive mechanism of action (Cai et al., 2018; Jiang and Li, 2020). And therefore, proteomics and metabolomics have been applied to explore SMI’s molecular mechanism. In our previous proteomics study, we found one of the major mechanisms of SMI protection against cardiac ischemia–reperfusion (IR) injury is modulation of the myocardial energy metabolism through multiple metabolic pathways (Zhan et al., 2015a). Chen et al. (Chen et al., 2015b) performed a metabolomic study on SMI’s treatment for doxorubicin-induced cardiomyopathy and suggested that it exerts cardioprotective effects by improving energy metabolism and attenuating oxidative stress. Although these studies helped to reveal some important mechanisms of SMI from systematic perspectives, the outcome was relatively simple and the proteomic and metabolic profiling were merely based on myocardial tissue analysis. Serum (including plasma) proteomics is attracting a growing study interest because of its large-scale and in-depth investigation of proteins from the blood system. Blood has a complex proteome that reflects many tissue proteome subsets (Liotta et al., 2003), and it can provide a much wider range of proteins than other single tissue or organ. Thus, serum proteomics has become a powerful tool for comprehensive mechanism exploring and biomarker discovery, especially in the area of cancer and cardiovascular diseases (Huang et al., 2017; Smith and Gerszten, 2017; Ku et al., 2020). Owing to the direct and inseparable contact between the blood and heart, blood system evidently embraces almost all of biological information from the whole heart, and it is conceivable to get much more and diverse proteomes in serum proteomics than in myocardial tissue proteomics for global molecular mechanism elaboration of SMI’s cardiovascular pharmacology. Nevertheless, the accessible serum biomarkers screened from serum proteomes will be of great benefit to SMI’s real-time effect detection for its preclinical and clinical evaluation in cardioprotection.

**FIGURE 1:**
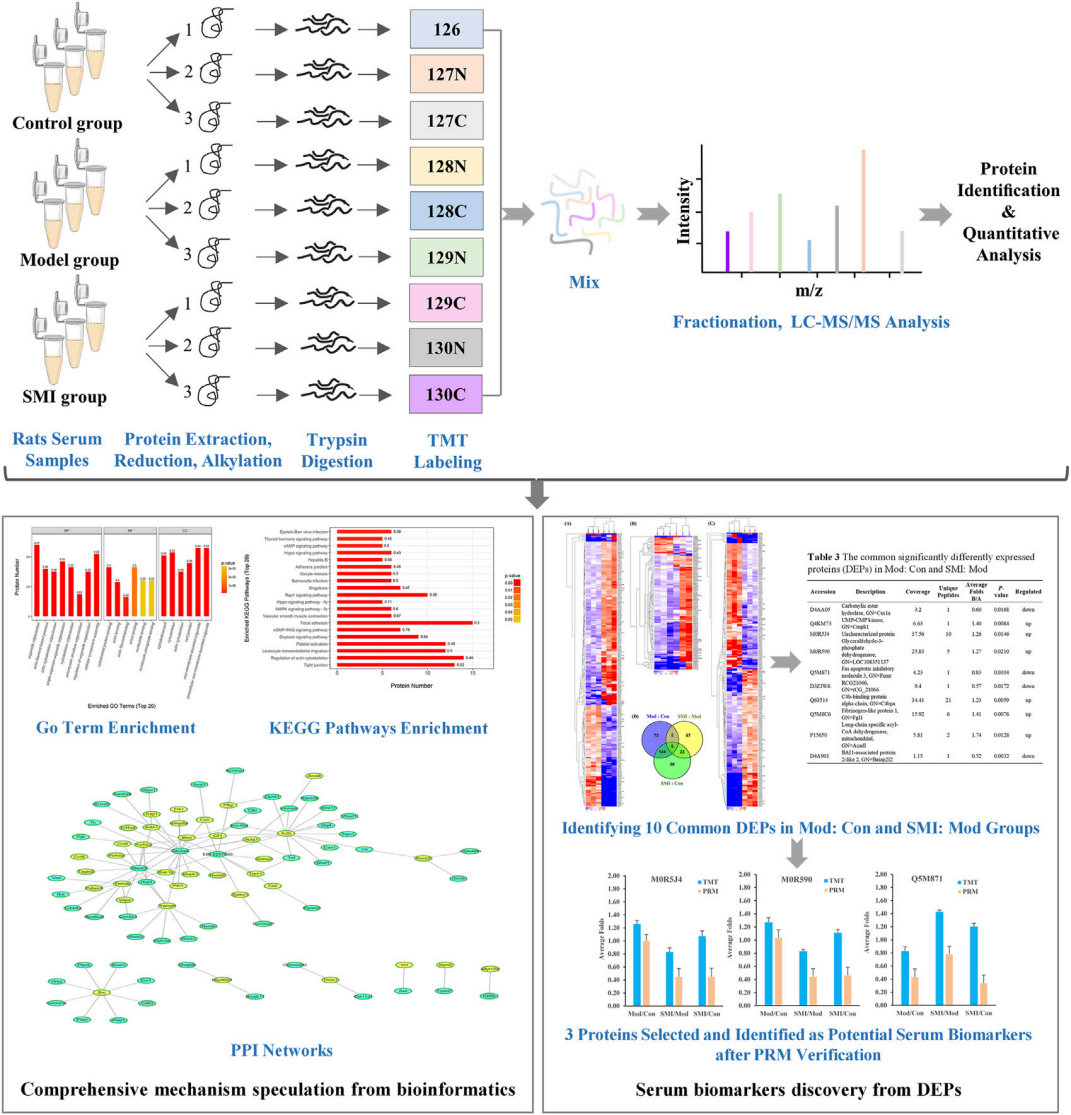
Workflow for exploring the comprehensive mechanism and identifying serum biomarkers of SMI’s cardioprotective effects.

In the present study, to explore the comprehensive mechanism and identify serum biomarkers of the SMI’s effect on myocardial ischemia, we used a tandem mass tag (TMT)–based quantitative proteomic method to detect the change in the serum protein profile in rats among the control group, isoproterenol (ISO)-induced myocardial ischemia group, and SMI intervention group. Figure 1 shows the workflow of the present TMT-based serum proteomic profiling and the investigation of differentially expressed serum proteins from ISO-induced myocardial ischemia rats with pretreatment of SMI vs non-pretreatment of SMI. This study would shed light on clarifying the global mechanism of SMI on ischemic heart disease and help reveal potential serum biomarkers for its cardioprotective effects.

## Materials and Methods

### SMI Preparation

SMI was provided by SZYY Group Pharmaceutical Limited (Jiangyan, Jiangsu, China). According to TCM Preparations’ standard of SMI (Standard Number: WS3-B-2865–98) from the Ministry of Health Drug Standards in China, 1,000 ml SMI is prepared from 100 g Panax ginseng C.A.Mey., 312 g Ophiopogon japonicus (Thunb.) Ker Gawl., and 156 g Schisandra chinensis (Turcz.) Baill.. In this study, high-performance liquid chromatography (HPLC) analysis was utilized to characterize its chemical composition *in vitro* and more than 12 compounds were identified based on standard substances (Supplementary Figure S1), which ensures batch-to-batch consistency of SMI. Based on the quantification of SMI by the HPLC method, the concentrations of 12 constituents such as ginsenoside Rg1, Re, Rf, Rb1, Rh1, Rc, Rb2, Rb3, Rd, (S)-ginsenoside Rg3, schisandrin, and schisandrol B were 110.3 ± 2.5, 128.4 ± 3.8, 46.9 ± 1.0, 262.8 ± 4.3, 104.0 ± 2.0, 223.8 ± 1.6, 134.7 ± 0.3, 11.5 ± 0.4, 106.1 ± 1.7, 18.8 ± 0.4, 27.6 ± 0.4, and 10.3 ± 0.1 μg/ml, respectively. Moreover, its principle components *in vivo* have been identified and quantified by high-performance liquid chromatography coupled with mass spectrometry in our previous study (Zhan et al., 2015b). It showed 11 constituents of ginsenoside Rg1, Re, Rf, Rg2, Rb1, Rd, Rc, ophiopogonin D, schisandrin, schisandrol B, and schizandrin B can be tracked in rat serum, and these active components should mainly contribute to SMI’s effects.

### Animals and Treatments

Adult male Sprague–Dawley (SD) rats, weighting 220–240 g, were purchased from the Experiment Animal Center of Zhejiang Province (Hangzhou, China). The rats were maintained under standard laboratory condition with temperature at 22 ± 2°C, relative humidity at 50 ± 10%, photo period (12 h dark/12 h light) and were fed normal diet and water ad libitum. After acclimatization of one week, all rats were randomly assigned into four groups (*n* = 10 per group) named control, model, high-dose SMI (SMI-high) and low-dose SMI (SMI-low) groups. SMI-high and SMI-low groups were given SMI (10.8 ml/kg/day) and SMI (5.4 ml/kg/day), respectively, which was calculated as rat doses in accordance with SMI’s clinical consumption. Control and model groups were given saline aqueous. All treatments were administrated intravenously by tail vein. After seven consecutive days of pretreatment, rats were intoxicated with ISO (85 mg/kg) by subcutaneous injection on two consecutive days (Song et al., 2016; Abdelrady et al., 2017), except for the control group which was given saline aqueous. The animal experiments (JUMC 2018–008) were performed in accordance with the Principles of Animal Care and Use approved by the Animal Ethic Review Committee of Jiaxing University.

### Sample Collection

Thirty minutes after the second ISO injection, all rats were anesthetized with 10% chloral hydrate (5 ml/kg) by intraperitoneal injection. Blood samples were drawn from the inferior vena cava and immediately transferred to the sterilized tubes. Immediately after the sacrifice of the rats, the hearts were removed and fixed in 10% formalin solution for histologic evaluation. The blood samples were centrifuged at 3,000×*g* for 10 min at 4°C. Serum was separated by collecting the supernatant and then divided into two parts. One part of serum samples was frozen immediately with liquid nitrogen and then stored at −80°C for proteomic analysis and verification, and the other part of serum samples was stored at 4°C for the real-time serum biochemical indicators detection.

### Serum LDH, CK-MB, SOD Assay, and Histology Analysis

Serum biochemical indicators of lactate dehydrogenase (LDH), creatine kinase-MB (CK-MB), and superoxide dismutase (SOD) release assay and histology analysis of heart tissue between groups were used to evaluate SMI’s effect on ISO-induced myocardial ischemia injury in rats. LDH, CK-MB, and SOD were detected using the colorimetric assay kits according to the manufacturers’ instructions (Nanjing Jiancheng Bioengineering Institute, Nanjing, China). Heart tissues were fixed in 4% neutral paraformaldehyde, paraffin-embedded, sliced into 4 µm sections, and stained with hematoxylin–eosin (H&E) staining. The histopathological changes were examined under the optical microscope (Leica DM3000LED, Germany).

### TMT-Based Serum Proteomic Analysis

#### TMT-Labeling and Fractionation

For TMT-labeling, serum pools at first were depleted of most abundant proteins using the Agilent Mouse 3 Multiple Affinity Removal System Column following the manufacturers’ protocol (Agilent Technologies). The samples were then digested using filter-aided sample preparation (FASP Digestion) according to the previous report (Wisniewski et al., 2009). The obtained peptides were estimated by UV light spectral density at 280 nm. Then, 100 μg of each peptide sample was labeled using the 10-plex TMT Mass Tagging kit (Thermo Fisher Scientific, Rockford, IL) according to the manufacturers’ instructions. In this study, three biological replications of each group for the control (Con), model (Mod), and SMI-high (SMI) were labeled with 126, 127N, 127C, 128N, 128C 129N, 129C, 130 N, and 130C, respectively. The TMT-labeled peptides from three groups were equivalently mixed, and then were fractionated into 10 fractions by an increasing acetonitrile step-gradient elution using pierce high-pH reversed-phase peptide fractionation kit (Thermo Fisher Scientific, Rockford, IL) according to the manufacturers’ instructions. The concentration of each fractionation sample was quantified at an absorbance of OD280 by UV light spectrum.

#### Data Acquisition for LC-MS/MS

Each fraction was injected for LC-MS/MS analysis. The peptide mixture was loaded onto a reverse phase trap column (Thermo Scientific Acclaim PepMap100, 100 μm × 2 cm, nanoViper C18) connected to the C18-reversed phase analytical column (Thermo Scientific EASY-Column, 10 cm × 75 μm, 3 μm, C18-A2) in buffer A (0.1% formic acid) and separated with a linear gradient of buffer B (84% acetonitrile and 0.1% formic acid) at a flow rate of 300 nL/min by IntelliFlow technology. The 90-min linear gradient was determined by the project proposal: 0–55% buffer B for 80 min, 55–100% buffer B for 5 min, and hold in 100% buffer B for 5 min.

LC-MS/MS analysis was performed on a Q-Exactive mass spectrometry (Thermo Scientific) that was coupled to Easy nLC (Proxeon Biosystems, now Thermo Fisher Scientific) for 90 min. The mass spectrometer was operated in positive ion mode. MS data were acquired using a data-dependent top 10 method dynamically choosing the most abundant precursor ions from the survey scan (300–1800 m/z) for HCD fragmentation. The automatic gain control (AGC) target was set to 3.0 × 10^–6^, and maximum inject time to 10 ms. Dynamic exclusion duration was 40 s. Survey scans were acquired at a resolution of 70,000 at m/z 200, and resolution for HCD spectra was set to 35,000 atm/z 200, and isolation width was 2 m/z. Normalized collision energy was 30 eV, and the underfill ratio, which specifies the minimum percentage of the target value likely to be reached at maximum fill time, was defined as 0.1%. The instrument was run with peptide recognition mode enabled.

#### Protein Identification and Quantitative Analysis

The MS/MS spectra data were searched using MASCOT engine (Matrix Science, London, United Kingdom) embedded into Proteome Discoverer 1.4 software (Thermo Scientific). The following parameters were set: all tryptic specificity was required; two missed cleavages were allowed; carbamidomethyl (C), TMT 10pLex (N-terminal), and TMT 10pLex (lysine, K) were set as the fixed modifications, the oxidation (methionine, M), and TMT 10pLex (tyrosine, Y) were set as the variable modifications; peptide mass tolerances were set at 20 ppm for all MS1 spectra acquired; and fragment mass tolerances were set at 0.1 Da for all MS2 spectra acquired. The false discovery rate (FDR) was set as ≤ 0.01. The protein ratios were calculated as the median of only unique peptides of the protein. All peptide ratios were normalized by the median protein ratio, and the median protein ratio should be “1” after the normalization. The upregulation threshold was set at the ratio of comparison groups >1.2 and *p*-value < 0.05, and the downregulation at the ratio of comparison groups <0.83 and *p*-value < 0.05.

#### Bioinformatics Analyses

Blast2GO (version 3.3.5) was used for the analysis of gene ontology (GO) annotation: blast searching, mapping, annotation, and InterProScan annotation. The identified proteins were classified based on the given protein’s molecular function, cellular component, and biological process. KEGG (Kyoto Encyclopedia of Genes and Genomes) pathway annotation was performed using KAAS (KEGG Automatic Annotation Server). The enrichment analyses for GO and KEGG annotations were performed using Fisher’s exact test, considering the whole quantified protein annotations as background dataset. Benjamini-Hochberg correction for multiple testing was further applied to adjust derived *p*-values. And only functional categories and pathways with *p*-values under a threshold of 0.05 were considered as significant. The protein-protein interaction (PPI) information of the studied proteins was retrieved from IntAct molecular interaction database (http://www.ebi.ac.uk/intact/) by their gene symbols or STRING software (http://string-db.org/). The results were downloaded in the XGMML format and imported into Cytoscape software (http://www.cytoscape.org/, version 3.2.1) to visualize and further analyze functional protein-protein interaction networks. Furthermore, the degree of each protein was calculated to evaluate the importance of the protein in the PPI network.

### Parallel Reaction Monitoring Verification

To verify the protein expression levels obtained by TMT analysis, the expression levels of selected proteins were further quantified by LC-PRMMS analysis (Peterson et al., 2012). In brief, peptides were prepared according to the TMT protocol, and an AQUA stable isotope peptide was spiked in each sample as internal standard reference. Tryptic peptides were loaded on C18 stage tips for desalting prior to reversed-phase chromatography on an Easy nLC-1200 system (Thermo Scientific). One-hour liquid chromatography gradients with acetonitrile ranging from 5 to 35% in 45 min were used. PRM analysis was performed on a Q-Exactive Plus mass spectrometer (Thermo Scientific). Methods optimized for collision energy, charge state, and retention times for the most significantly regulated peptides were generated experimentally using unique peptides of high intensity and confidence for each target protein. The mass spectrometer was operated in positive ion mode and with the following parameters: the full MS1 scan was acquired with the resolution of 70,000 (at 200 m/z), automatic gain control (AGC) target values 3.0 × 10^–6^, and a 250 ms maximum ion injection times. Full MS scans were followed by 20 PRM scans at 35,000 resolution (at m/z 200) with AGC 3.0 × 10^–6^ and maximum injection time 200 ms. The targeted peptides were isolated with a 2Th window. Ion activation/dissociation was performed at normalized collision energy of 27 in a higher-energy dissociation (HCD) collision cell. The raw data were analyzed using Skyline (MacCoss Lab, University of Washington) (MacLean et al., 2010), where signal intensities for individual peptide sequences for each of the significantly altered proteins were quantified relative to each sample and normalized to standard reference.

### Statistics

Data were expressed as mean ± standard deviation (SD). Statistical analysis was performed using SPSS software (version 16.0). Statistical differences between two groups were analyzed using Student’s t-test with significant difference at *p* < 0.05.

## Results

### Cardioprotective Effect of SMI on ISO-Induced Myocardial Ischemia Injury in Rats

**FIGURE 2:**
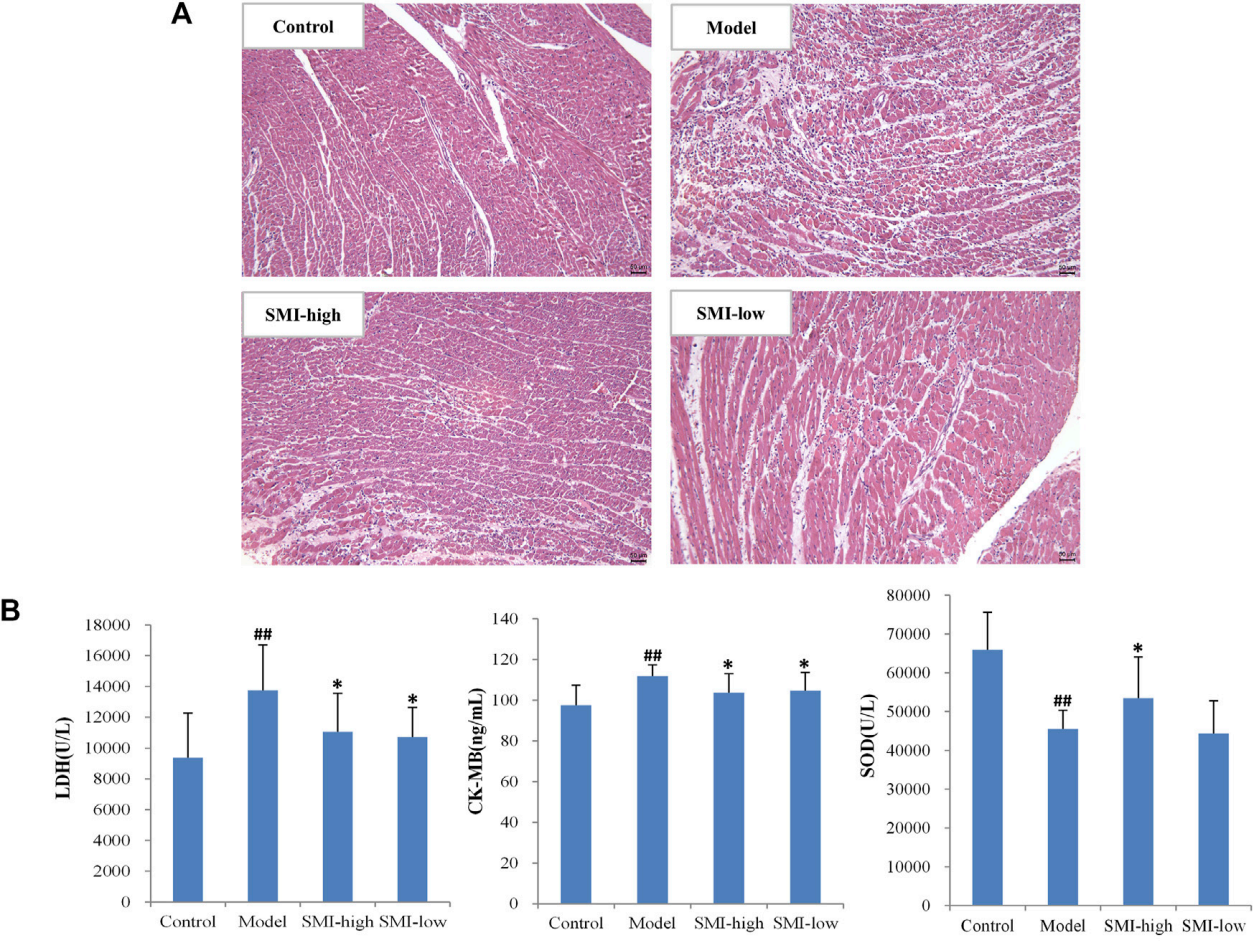
Cardioprotective effect of SMI on ISO-induced myocardial ischemia injury in rats. **(A)** Representative H&E images (200×) of myocardial histology analysis for control, model, SMI-high, and SMI-low group rats. **(B)** Serum LDH, CK-MB, and SOD levels in control, model, SMI-high, and SMI-low group rats. (Data were expressed as Mean ± SD (*n* = 10), ^##^ *p* < 0.01 vs. control, ^*^ *p* < 0.05 vs. model).

To confirm the cardioprotective effect of SMI on ischemia injury in rats, the histology analysis and myopathological enzymatic indicators detection of LDH, CK-MB, and SOD in serum were performed before serum proteomic study. The representative images of myocardial ischemia section in control, model, SMI-high, and SMI-low groups exhibited SMI pretreatment rats modestly reduced infiltration of inflammatory cells and fibrotic phenomenon compared to model group rats (Figure 2A). It is found that serum LDH and CK-MB levels significantly increased and the SOD level significantly decreased in rats after ISO-induced myocardial ischemia injury, while SMI pretreatment could significantly attenuate the changes of these enzymatic indicators (Figure 2B).

### Quantitative Identification of Differentially Expressed Serum Proteins

**FIGURE 3:**
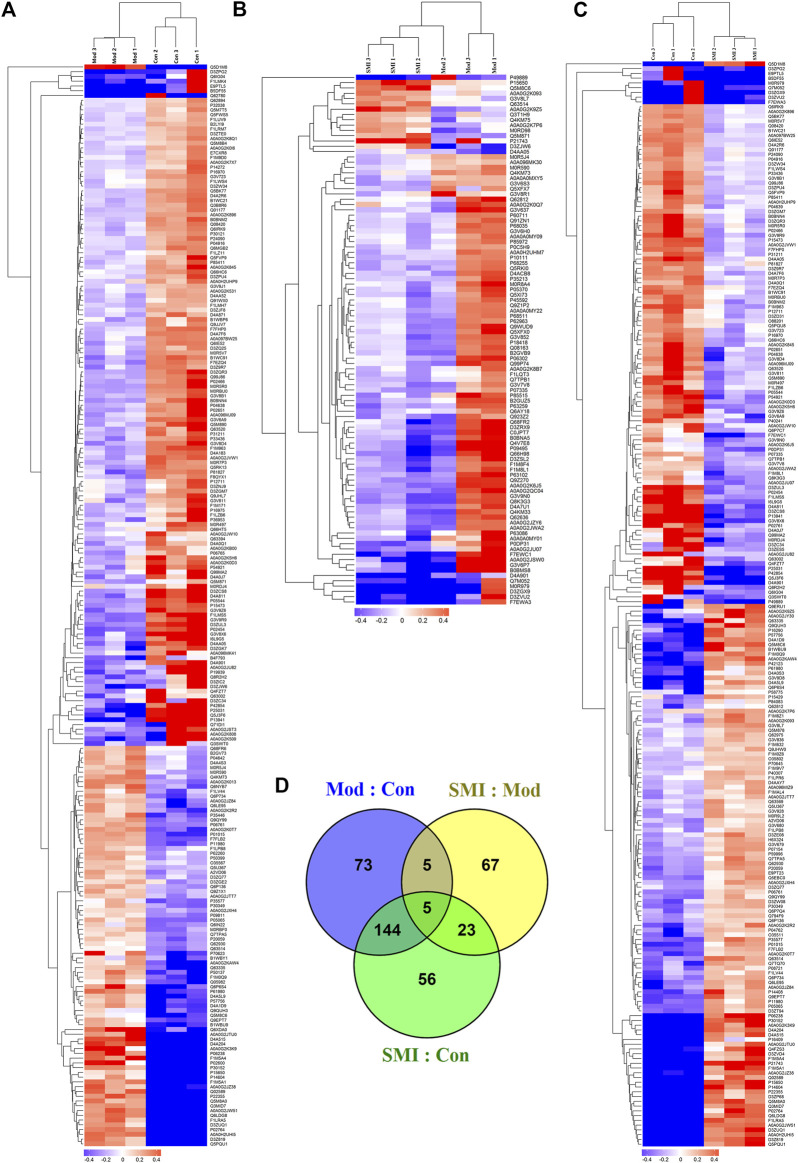
Differentially expressed proteins (DEPs) profiles by TMT-based proteomic analysis. **(A, B, C)** Heat maps of the overall differential concentrations of proteins quantified in Mod: Con **(A)**, SMI: Mod **(B)**, and SMI: Con **(C)**, respectively. **(D)** Venn diagram showing overlap of all DEPs among Mod: Con, SMI: Mod, and SMI: Con.

In order to globally understand the molecular mechanisms and reveal serum biomarkers of SMI’s cardioprotective effect on ISO-induced acute myocardial ischemia in rats, a TMT-based serum proteomic analysis was carried out. After merging data from the respective replicates, 1,009 proteins were identified, of which 558 contained at least two unique peptides. Moreover, 1,000 proteins were quantified in Mod: Con, SMI: Mod, and SMI: Con, respectively. Further analysis identified that there were 227 (85 upregulated, 142 downregulated), 100 (14 upregulated, 86 downregulated), and 228 (115 upregulated, 113 downregulated) significantly differentially expressed proteins (DEPs) in Mod: Con, SMI: Mod, and SMI: Con, respectively (Table 1 and Supplementary Table S1). The gene names, accession numbers, and relative abundance ratios of the upregulated and downregulated proteins of SMI: Mod are listed in Table 2. The overall differential concentrations of proteins between groups were visualized using the global heat map analysis (Figures 3A–C). The Venn diagram (Figure 3D) showed the overlap of all DEPs between the three groups. It found 10 common DEPs in Mod: Con and SMI: Mod (Table 3) which might deserve special focus because their change was simultaneously related to ISO-induced myocardial ischemia and SMI’s intervention effect.

**TABLE 1:** Number of differentially expressed proteins (DEPs) identified among groups in TMT-based proteomic analysis.

Comparisons	Quantified	Upregulated	Downregulated	Total difference
Mod/Con	1,000	85	142	227
SMI/Mod	1,000	14	86	100
SMI/Con	1,000	115	113	228

**TABLE 2:** Significantly up- and downregulated serum proteins in SMI: Mod group rats.

Accession	Description	Coverage	Unique peptides	Average folds SMI/Mod	*p*-value
D3ZJW6	RCG21066, GN = rCG_21,066	9.40	1	1.59	0.0285
A0A0G2K9Z5	Uncharacterized protein	9.32	1	1.56	0.0284
Q5M871	Fas apoptotic inhibitory molecule 3, GN = Fcmr	4.23	1	1.43	0.0343
M0RD98	Uncharacterized protein	6.56	1	1.33	0.0481
Q5M8C6	Fibrinogen-like protein 1, GN = Fgl1	15.92	6	1.32	0.0082
A0A0G2K7P6	Uncharacterized protein	36.17	3	1.30	0.0076
P15650	Long-chain specific acyl-CoA dehydrogenase, mitochondrial, GN = Acadl	5.81	2	1.29	0.0429
P21743	Insulin-like growth factor–binding protein 1, GN = Igfbp1	5.15	2	1.29	0.0124
G3V8L7	Integrin alpha M, GN = Itgam	0.61	1	1.24	0.0128
Q4KM75	CD5 antigen-like, GN = Cd5l	39.60	17	1.22	0.0492
Q3T1H9	Similar to Rnf37-pending protein, GN = Ubox5	1.86	1	1.21	0.0106
A0A0G2K093	Heat-shock 70-kDa protein 13, GN = Hspa13	3.01	1	1.21	0.0379
D4AA05	Carboxylic ester hydrolase, GN = Ces1a	3.20	1	1.21	0.0377
Q63514	C4b-binding protein alpha chain, GN = C4bpa	34.41	21	1.21	0.0328
A0A0G2K0Q7	Myosin light-chain kinase, GN = Mylk	1.74	3	0.83	0.0416
G3V7V8	Phosphodiesterase, GN = Pde5a	10.32	5	0.83	0.0436
A0A096MK30	Moesin, GN = Msn	14.73	9	0.83	0.0338
M0R5J4	Uncharacterized protein	37.56	10	0.83	0.0447
M0R590	Glyceraldehyde-3-phosphate dehydrogenase, GN = LOC108351137	25.83	5	0.83	0.0095
P62963	Profilin-1, GN = Pfn1	67.14	7	0.83	0.0339
F1LQT3	Rho-associated protein kinase, GN = Rock2	0.44	1	0.82	0.0207
P18418	Calreticulin, GN = Calr	28.13	11	0.82	0.0340
P07335	Creatine kinase B-type, GN = Ckb	22.83	6	0.82	0.0035
Q7TPB1	T-complex protein 1 subunit delta, GN = Cct4	2.04	1	0.82	0.0086
P60711	Actin, cytoplasmic 1, GN = Actb	53.33	1	0.82	0.0218
P45592	Cofilin-1, GN = Cfl1	50.00	7	0.82	0.0399
A0A0G2K8B7	Eukaryotic initiation factor 4 A-II, GN = Eif4a2	2.21	1	0.82	0.0463
Q9Z1P2	Alpha-actinin-1, GN = Actn1	30.72	14	0.82	0.0387
A0A0A0MXY5	ATP-dependent 6-phosphofructokinase, GN = Pfkp	2.00	1	0.81	0.0252
Q62812	Myosin-9, GN = Myh9	24.58	1	0.81	0.0006
A0A0G2JZY6	Spectrin beta chain, GN = Sptbn1	3.42	7	0.81	0.0097
P0C5H9	Mesencephalic astrocyte-derived neurotrophic factor, GN = Manf	4.47	1	0.81	0.0150
P10111	Peptidyl-prolyl *cis*-trans isomerase A, GN = Ppia	53.05	9	0.81	0.0093
A0A0A0MY22	Sialate O-acetylesterase, GN = Siae	1.29	1	0.81	0.0196
Q6AY18	SAR1 gene homolog a (*S. cerevisiae*), isoform CRA_b, GN = Sar1a	4.04	1	0.81	0.0484
D4ACB8	Chaperonin subunit 8 (theta) (predicted), isoform CRA_a, GN = Cct8	4.01	2	0.81	0.0045
A0A0H2UHM7	Tubulin alpha chain, GN = LOC100909441	33.26	3	0.80	0.0080
P35213	14-3–3 protein beta/alpha, GN = Ywhab	26.42	2	0.80	0.0013
P85972	Vinculin, GN = Vcl	56.94	59	0.80	0.0291
Q5XFX7	RAD23 homolog a (*S. cerevisiae*), GN = Rad23a	2.56	1	0.80	0.0067
G3V6H0	RAB1B, member RAS oncogene family like, GN = LOC100363782	11.94	1	0.80	0.0186
Q4KM73	UMP-CMP kinase, GN = Cmpk1	6.63	1	0.79	0.0204
P68255	14-3–3 protein theta, GN = Ywhaq	30.61	2	0.79	0.0092
A0A0A0MY09	Endoplasmin, GN = Hsp90b1	9.08	7	0.79	0.0403
G3V852	RCG55135, isoform CRA_b, GN = Tln1	44.51	95	0.79	0.0494
Q99P74	Ras-related protein Rab-27B, GN = Rab27 b	5.05	1	0.79	0.0292
P06302	Prothymosin alpha, GN = Ptma	11.61	1	0.79	0.0123
Q7M052	Alpha-1-macroglobulin	12.50	1	0.79	0.0092
Q9WUD9	Proto-oncogene tyrosine-protein kinase src, GN = Src	4.66	3	0.78	0.0404
G3V8R1	Nucleobindin 2, isoform CRA_b, GN = Nucb2	2.62	1	0.78	0.0425
P68035	Actin, alpha cardiac muscle 1 OS = *Rattus norvegicus*, GN = Actc1	44.30	4	0.78	0.0107
Q08163	Adenylyl cyclase–associated protein 1, GN = Cap1	38.61	14	0.77	0.0239
P68511	14-3–3 protein eta, GN = Ywhah	44.72	6	0.77	0.0287
Q5XFX0	Transgelin-2, GN = Tagln2	71.36	11	0.77	0.0299
Q5RKI0	WD repeat-containing protein 1, GN = Wdr1	19.80	11	0.77	0.0076
P05370	Glucose-6-phosphate 1-dehydrogenase, GN = G6pdx	9.51	5	0.77	0.0355
B2GVB9	Fermitin family member 3, GN = Fermt3	23.91	14	0.77	0.0293
M0R8A4	Microtubule-associated protein RP/EB family member 2, GN = Mapre2	18.22	3	0.77	0.0316
Q62636	Ras-related protein Rap-1b, GN = Rap1b	32.07	7	0.77	0.0222
D4A901	Bai1-associated protein 2-like 2, GN = Baiap2l2	1.15	1	0.76	0.0192
Q4KM33	Pleckstrin, GN = Plek	37.71	10	0.76	0.0332
Q91ZN1	Coronin-1a, GN = Coro1a	23.43	11	0.76	0.0064
A0A0G2JWA2	Phosphoinositide phospholipase C, GN = Plcg2	0.71	1	0.76	0.0185
G3V6S3	Calumenin, GN = Calu	11.75	3	0.75	0.0022
D4A7U1	Zyxin, GN = Zyx	6.74	3	0.75	0.0102
Q923Z2	Tropomyosin 1, alpha, isoform CRA_a, GN = Tpm1	31.34	3	0.75	0.0229
Q5XI73	Rho GDP-dissociation inhibitor 1, GN = Arhgdia	32.84	7	0.74	0.0232
F1M8F4	Glia maturation factor gamma, GN = Gmfg	9.86	1	0.74	0.0259
A0A0G2QC04	Plastin 1, GN = Pls1	9.37	4	0.73	0.0026
D3ZRX9	Calponin, GN = Cnn2	3.64	1	0.73	0.0256
C0JPT7	Filamin A, GN = Flna	36.98	75	0.73	0.0329
P63259	Actin, cytoplasmic 2, GN = Actg1	53.33	1	0.73	0.0092
P63086	Mitogen-activated protein kinase 1, GN = Mapk1	1.96	1	0.73	0.0475
P63102	14-3–3 protein zeta/delta, GN = Ywhaz	55.10	7	0.73	0.0175
B0BNA5	Coactosin-like protein, GN = Cotl1	52.11	7	0.73	0.0277
G3V9N0	Polyadenylate-binding protein, GN = Pabpc4	0.93	1	0.73	0.0131
G3V637	Syntaxin-binding protein 2, isoform CRA_b, GN = Stxbp2	1.68	1	0.72	0.0034
B2GUZ5	F-actin-capping protein subunit alpha-1, GN = Capza1	5.24	1	0.72	0.0052
A0A0G2K6J5	Myosin light polypeptide 6, GN = Myl6	51.66	6	0.72	0.0141
M0R979	Thrombospondin 1, GN = Thbs1	52.36	1	0.71	0.0193
F1M8L1	Kinesin-like protein, GN = Kif2a	1.14	1	0.71	0.0212
Q9Z270	Vesicle-associated membrane protein-associated protein A, GN = Vapa	6.83	1	0.71	0.0053
Q68FR2	Bridging integrator 2, GN = Bin2	24.85	11	0.71	0.0447
A0A0G2JU07	Ubiquitin-conjugating enzyme E2 variant 2, GN = Ube2v2	6.90	1	0.71	0.0025
Q8K3G3	Four and a half LIM domains 1, isoform CRA_e, GN = Fhl1	3.81	1	0.71	0.0068
P85515	Alpha-centractin, GN = Actr1a	3.46	1	0.69	0.0163
D3ZSL2	ABRA C-terminal-like, GN = Abracl	30.86	2	0.68	0.0169
F7EWA3	SP140 nuclear body protein, GN = Sp140	0.91	1	0.68	0.0370
P09495	Tropomyosin alpha-4 chain, GN = Tpm4	59.68	8	0.67	0.0242
Q4V7E8	Leucine-rich repeat flightless-interacting protein 2, GN = Lrrfip2	2.06	1	0.67	0.0163
Q66H98	Serum deprivation-response protein, GN = Sdpr	5.76	3	0.66	0.0068
A0A0A0MY01	Fatty acid-binding protein, intestinal, GN = Fabp2	6.06	1	0.66	0.0181
P0DP31	Calmodulin-3, GN = Calm3	30.20	4	0.65	0.0119
D3ZVU2	FANCD2 and FANCI-associated nuclease 1, GN = Fan1	0.69	1	0.65	0.0404
G3V6P7	Myosin, heavy polypeptide 9, nonmuscle, GN = Myh9	24.44	1	0.63	0.0278
B0BMS8	Myl9 protein, GN = Myl9	31.40	1	0.62	0.0230
D3ZGX9	Sema domain, immunoglobulin domain (ig), short basic domain, secreted (semaphorin), 3 F (predicted), isoform CRA_a, GN = Sema3f	0.76	1	0.61	0.0127
F7EWC1	Vasodilator-stimulated phosphoprotein, GN = Vasp	5.88	2	0.58	0.0046
A0A0G2JSW0	Myosin regulatory light chain 12B, GN = Myl12 b	31.40	1	0.58	0.0167
P49889	Estrogen sulfotransferase, isoform 3, GN = Ste	2.37	1	0.54	0.0072

**TABLE 3:** Common significantly differentially expressed proteins (DEPs) in Mod: Con and SMI: Mod.

Accession	Description	Coverage	Unique peptides	Average folds mod/Con	p-value	Regulated	Average folds SMI/Mod	p-value	Regulated
D4AA05	Carboxylic ester hydrolase, GN = Ces1a	3.2	1	0.60	0.0168	Down	1.21	0.0377	Up
Q4KM73	UMP-CMP kinase, GN = Cmpk1	6.63	1	1.40	0.0084	Up	0.79	0.0204	Down
M0R5J4	Uncharacterized protein	37.56	10	1.26	0.0146	Up	0.83	0.0447	Down
M0R590	Glyceraldehyde-3-phosphate dehydrogenase, GN = LOC108351137	25.83	5	1.27	0.0210	Up	0.83	0.0095	Down
Q5M871	Fas apoptotic inhibitory molecule 3, GN = Fcmr	4.23	1	0.83	0.0104	Down	1.43	0.0343	Up
D3ZJW6	RCG21066, GN = rCG_21,066	9.4	1	0.57	0.0172	Down	1.59	0.0285	Up
Q63514	C4b-binding protein alpha chain, GN = C4bpa	34.41	21	1.23	0.0059	Up	1.21	0.0328	Up
Q5M8C6	Fibrinogen-like protein 1, GN = Fgl1	15.92	6	1.41	0.0076	Up	1.32	0.0082	Up
P15650	Long-chain specific acyl-CoA dehydrogenase, mitochondrial, GN = Acadl	5.81	2	1.74	0.0128	Up	1.29	0.0429	Up
D4A901	Bai1-associated protein 2-like 2, GN = Baiap2l2	1.15	1	0.52	0.0032	Down	0.76	0.0192	Down

### Bioinformatics Analysis of DEPs

#### Gene Ontology (GO) Analysis

**FIGURE 4:**
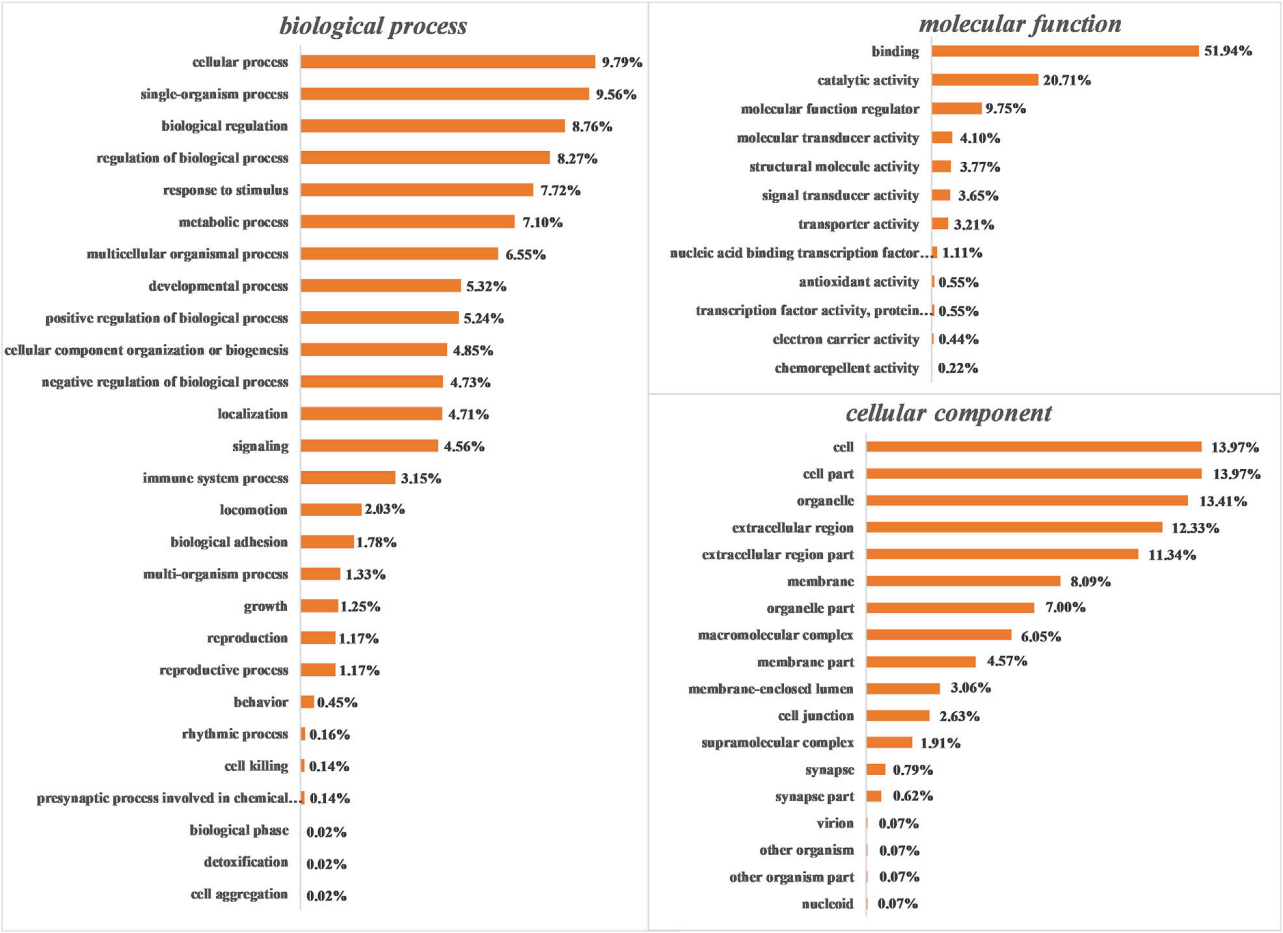
Gene ontology (GO) analysis of all differentially expressed proteins (DEPs). Proteins were classified according to biological process (BP), molecular function (MF), and cellular component (CC) by Blast2GO software (version 3.3.5).

GO analysis was performed to functionally classify the DEPs, with the proteins categorized according to biological process (BP), cellular components (CC), and molecular functions (MF) using Blast2GO software (version 3.3.5). As shown in Figure 4, proteins were separated according to their BP into 26 subcategories that included the following: cellular process (9.97%), single-organism process (9.56%), biological regulation (8.76%), regulation of biological process (8.27%), response to stimulus (7.72%), and metabolic process (7.10%), among others. The four major MF subcategories were as follows: binding (51.94%), catalytic activity (20.71%), molecular function regulator (9.75%), and molecular transducer activity (4.10%). The eight main CC subcategories were as follows: cell (13.97%), cell part (13.97%), organelle (13.41%), extracellular region (12.33%), extracellular region part (11.34%), membrane (8.09%), organelle part (7.00%), and macromolecular complex (6.05%).

**FIGURE 5:**
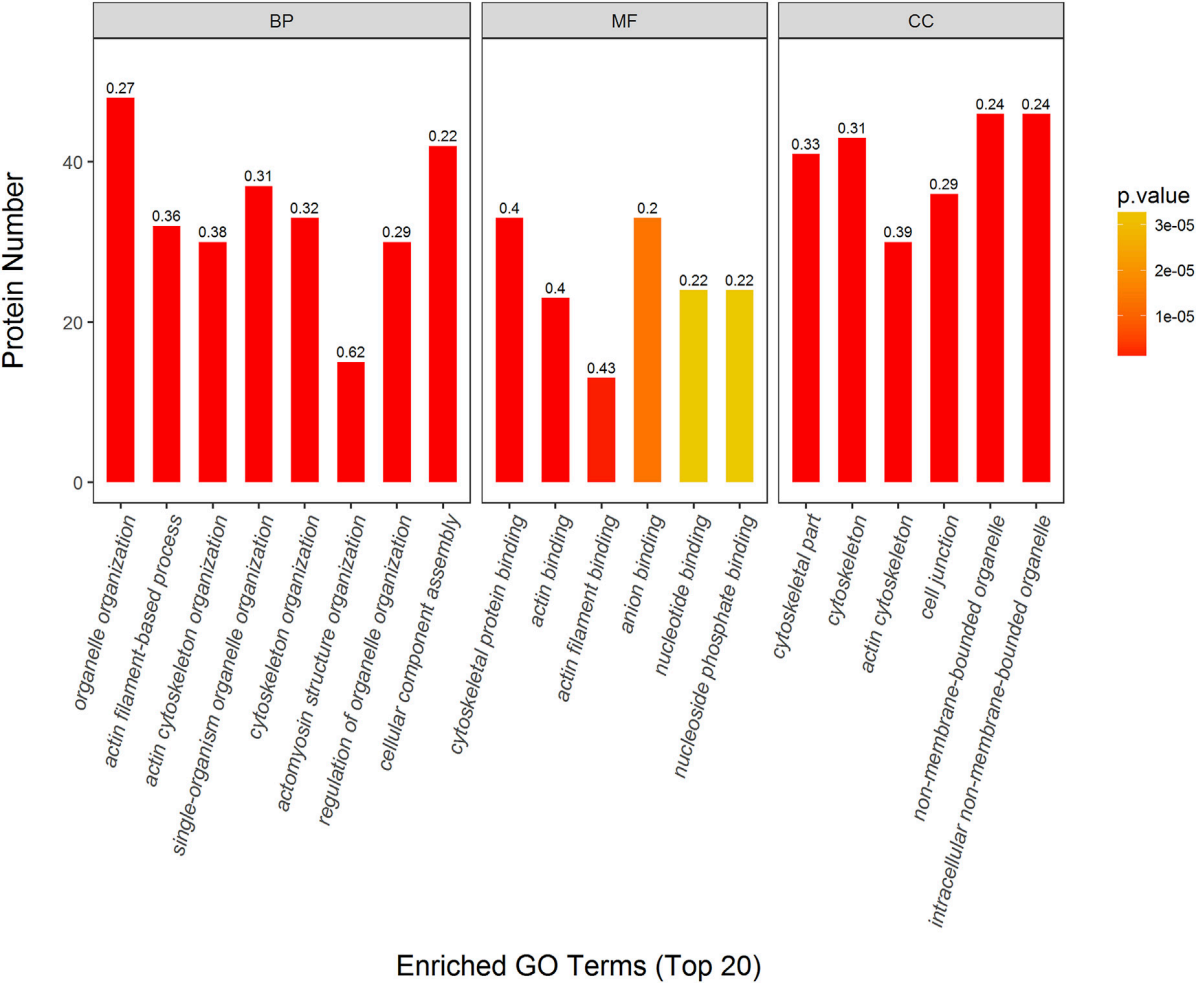
Top 20 enriched GO terms of significantly differentially expressed proteins (DEPs) in SMI: Mod. *p* value (more lower more intense in red color) represents the enriched degree. Each number above the bar charts is the richFactor (richFactor ≤1), which is the ratio of the number of proteins that have been annotated in this category.

The DEPs were further characterized in Mod: Con, SMI: Mod, and SMI: Con, respectively, using GO enrichment analysis. Figure 5 shows the top 20 enriched GO terms in SMI: Mod. The top three enriched BP categories were organelle organization (48 proteins, *p* = 6.35*e^−14^, richFactor = 0.27), actin filament-based process (32 proteins, *p* = 1.10*e^−12^, richFactor = 0.36), and actin cytoskeleton organization (30 proteins, *p* = 1.92*e^−12^, richFactor = 0.38). The top three enriched MF categories were cytoskeletal protein binding (33 proteins, *p* = 1.29*e^−14^, richFactor = 0.40), actin binding (23 proteins, *p* = 3.52*e^−10^, richFactor = 0.40), and actin filament binding (13 proteins, *p* = 1.16*e^−6^, richFactor = 0.43). The top three enriched CC categories were cytoskeletal part (41 proteins, *p* = 7.49*e^−15^, richFactor = 0.33), cytoskeleton (43 proteins, *p* = 7.56*e^−15^, richFactor = 0.31), and actin cytoskeleton (30 proteins, *p* = 3.78*e^−13^, richFactor = 0.39). For Mod: Con and SMI: Con group, the top 20 enriched GO terms are illustrated in Supplementary Figure S2, S3, respectively.

#### KEGG Pathway Analysis

**FIGURE 6:**
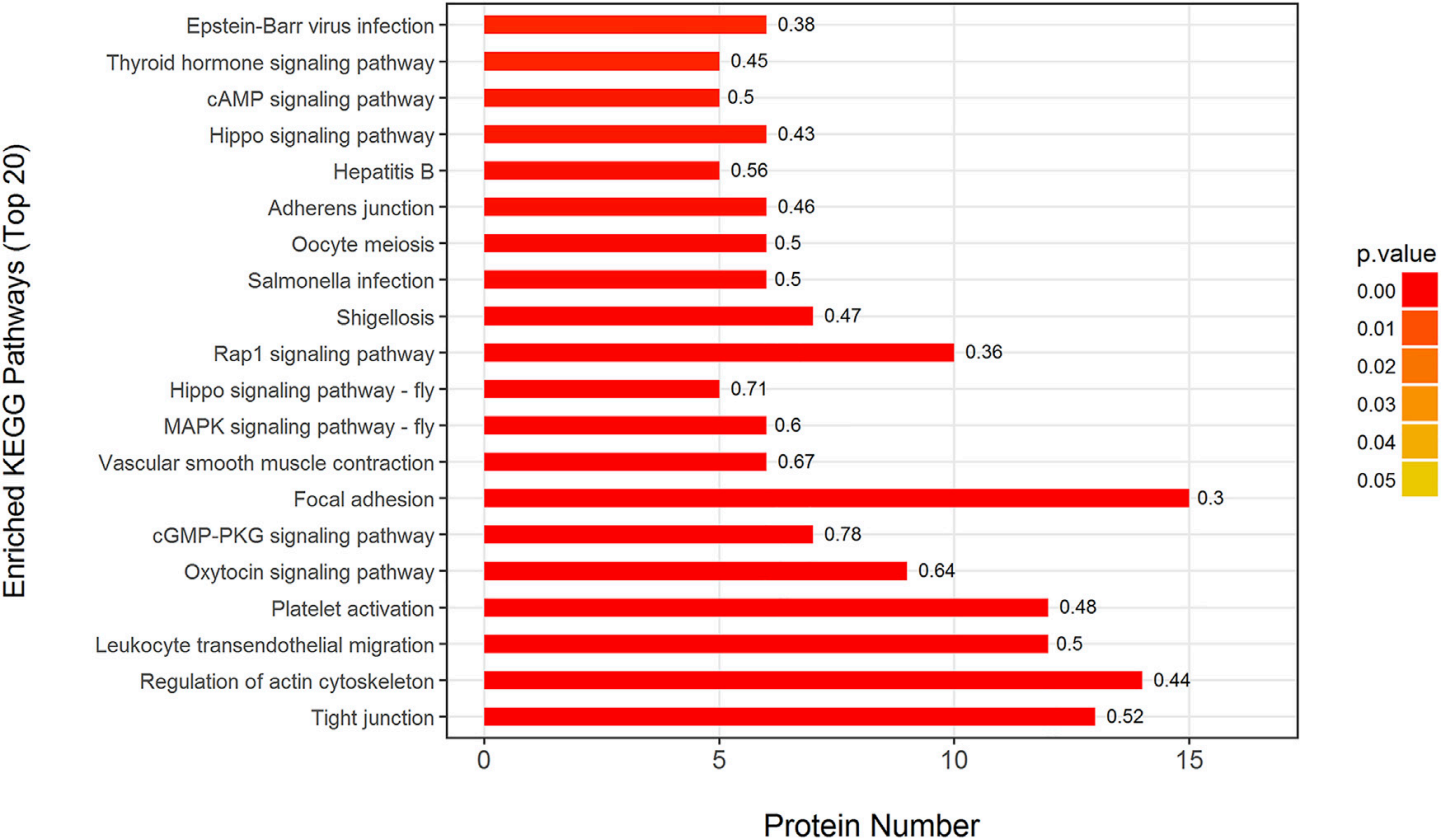
Top 20 enriched KEGG pathways in SMI: Mod. *p* value (more lower more intense in red color) represents the enriched degree. Each number above the bar charts is the richFactor (richFactor ≤1), which is the ratio of the number of proteins that have been annotated in this category.

To analyze the biological pathways that responded to ISO-induced myocardial ischemia and SMI’s intervention effect, all DEPs were further analyzed in Mod: Con, SMI: Mod, and SMI: Con, respectively, using the KEGG database. It was found that 227 DEPs in Mod: Con can be mapped to 200 signaling pathways and three pathways of protein digestion and absorption, spliceosome and fatty acid degradation were significantly enriched (*p* < 0.05), and 228 DEPs in SMI: Con can be mapped to 197 signaling pathways and three pathways of vitamin digestion and absorption, NF-kappa B signaling pathway and fatty acid degradation were significantly enriched (*p* < 0.05) (Supplementary Figure S4). Interestingly, we found that 100 DEPs in SMI: Mod can be mapped to 184 signaling pathways and 47 of them were significantly enriched (*p* < 0.05, Supplementary Table S2). Figure 6 shows the top 20 enriched pathways in SMI: Con which include tight junction, regulation of actin cytoskeleton, leukocyte transendothelial migration, platelet activation, oxytocin signaling pathway, cGMP-PKG signaling pathway, focal adhesion, vascular smooth muscle contraction, MAPK signaling pathway-fly, and Rap1 signaling pathway, among others.

#### Protein–Protein Interaction Network

**FIGURE 7:**
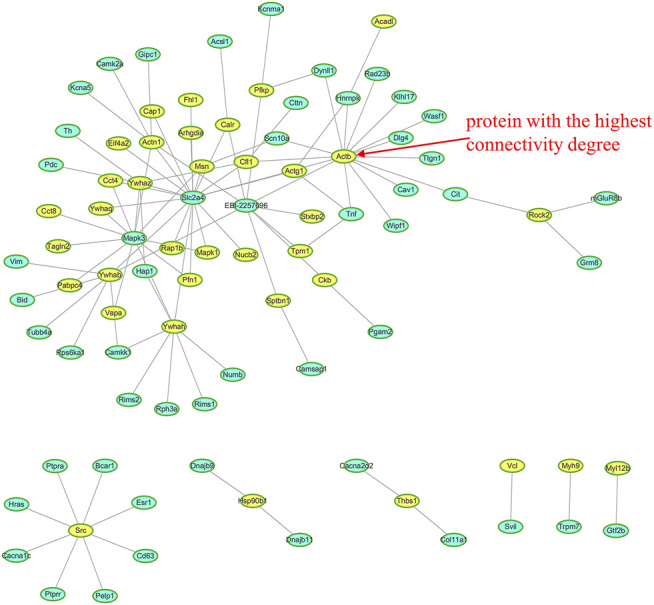
Seven protein-protein interaction (PPI) networks in SMI: Mod. The yellow node represents the differentially expressed proteins (DEPs), and the blue node represents the nonchanged protein. The most complex network involves 30 DEPs involved and Actb (ProteinID-P60711) has the highest connectivity degree of 15.

Protein-protein interaction (PPI) network was constructed for all DEPs to reveal the association of these DEPs’ function. The results show that DEPs can be mapped to known 9, 7, and 7 PPI networks in Mod: Con, SMI: Mod, and SMI: Con, respectively. The 7 PPI networks of DEPs in SMI: Mod are shown in Figure 7, where the yellow nodes represent DEPs found in this study. In the most complex network, there is 30 DEPs involved. One of the lead proteins, Actb (ProteinID-P60711), which is actin with the highest connectivity degree of 15, and this protein is also found involved in the top five KEGG pathways (tight junction, regulation of actin cytoskeleton, leukocyte transendothelial migration, platelet activation, and oxytocin signaling pathway). The other four lead proteins were Ywhah (ProteinID-P68511; connectivity degree: 8 proteins), Src (ProteinID-Q9WUD9; connectivity degree: 8 proteins), Ywhab (ProteinID-P35213; connectivity degree: 7 proteins), and Actn1 (ProteinID-Q9Z1P2; connectivity degree: 6 proteins). For Mod: Con, the PPI networks are illustrated in Supplementary Figure S5.

### PRM Verification

**FIGURE 8:**
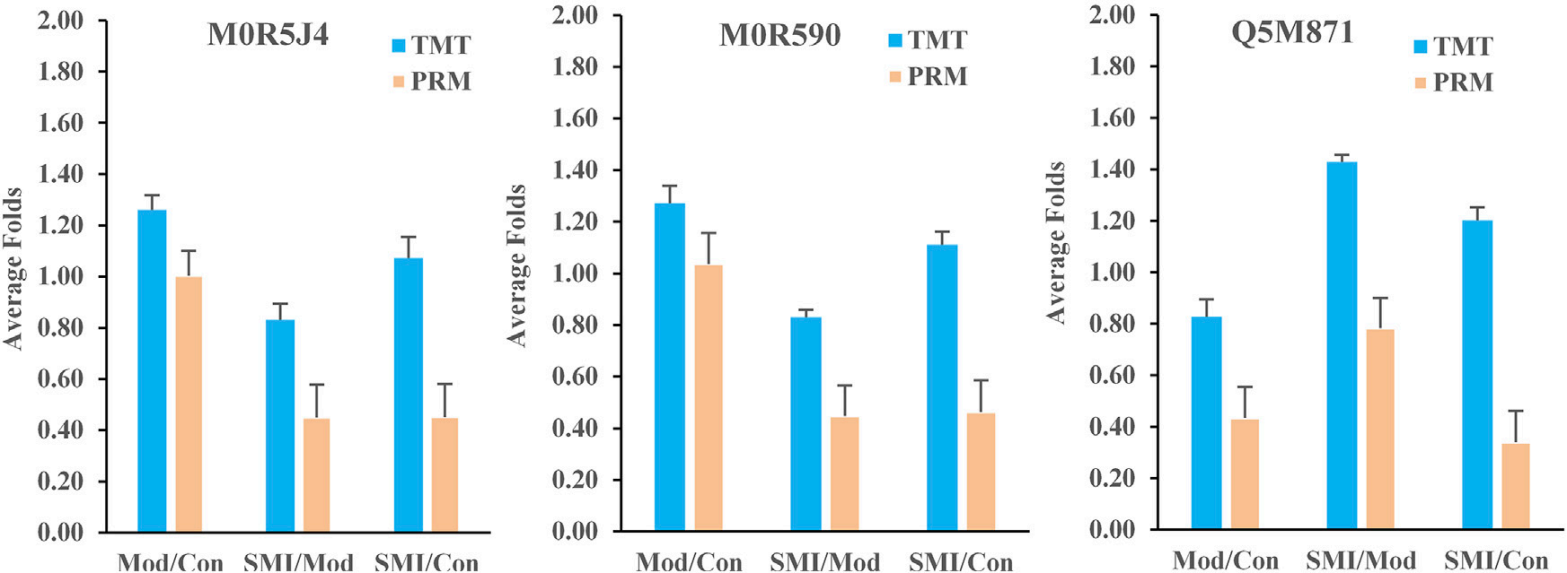
Average protein relative expression folds of the three proteins (M0R5J4, M0R590, and Q5M871) in Mod: Con, SMI: Mod, and SMI: Con by TMT and PRM, respectively. *n* = 3 samples for each group (3 data points for each bar), each consisting of serum of three rats.

To further verify the results of TMT-based proteomics, three DEPs (ProteinID: M0R5J4, M0R590, and Q5M871) in Table 3 were selected for PRM analysis. The screening criteria were formulated based on the following principles: 1) the common serum proteins which both were regulated in ISO-induced myocardial ischemia and SMI’s intervene; 2) proteins which were regulated in ISO-induced myocardial ischemia rats serum while were back-regulated in SMI pretreatment rats serum; 3) proteins with unique peptides of greater than one, as identified by TMT. After normalized, the results of the relative quantitative expression (Figure 8) showed that the three proteins exhibited similar trends to those observed in the TMT results, which supports the plausibility and reliability of the proteomics data.

## Discussion

SMI is an important Chinese herbal medicine for treating ischemic heart diseases, and its clinical benefits have been found in patients with acute coronary syndrome or chronic heart failure (Zhang et al., 2008; Wang et al., 2020). Isoproterenol (ISO), a synthetic catecholamine and *β*-adrenergic agonist, is usually used to set up standardized myocardial ischemia injury model in rats. In our previous study, we have performed pharmacodynamical evaluation of SMI and its main components of ginsenoside Rg1, ginsenoside Rb1, and schizandrin on ISO-induced rat myocardial ischemia (Zhan et al., 2014; Zhan et al., 2018). In this study, the investigation of histopathology indicated ISO-induced myocardial injury was significantly alleviated with SMI pretreatment, and moreover, the regulation on serum cardiac biomarkers of LDH, CK-MB, and SOD also demonstrated SMI’s anti-inflammatory and anti-oxidation activities. Taken together, these studies evidenced cardioprotective effect of SMI on ISO-induced myocardial ischemia injury.

Using 2-dimensional gel electrophoresis method, our previous proteomic study detected the changes of 14 proteins in myocardial tissue of IR injury rats after SMI treatment, and most of these altered proteins were related to energy metabolism including glucose metabolism, lipid metabolism, TCA cycle, and respiratory chain (Zhan et al., 2015a). With the purpose of comprehensive understanding SMI’s molecular mechanism, in this quantitative serum proteomic analysis, we determined 100 DEPs (much more than previous myocardial proteome) in a serum of myocardial ischemia rats that ascribe to SMI’s intervention, and these proteins are much different from previous founds. There are six DEPs including long-chain specific acyl-CoA dehydrogenase (ProteinID-P15650), glyceraldehyde-3-phosphate dehydrogenase (ProteinID-M0R590), creatine kinase B-type (ProteinID-P07335), ATP-dependent 6-phosphofructokinase (ProteinID-A0A0A0MXY5), sialate O-acetylesterase (ProteinID-A0A0A0MY22), and glucose-6-phosphate 1-dehydrogenase (ProteinID-P05370) detected in serum proteome which are related to energy metabolism. Moreover, two of them (long-chain specific acyl-CoA dehydrogenase and creatine kinase B-type) are the same enzyme but different subtypes (short-chain specific acyl-CoA dehydrogenase and creatine kinase M-type) as found in myocardial proteome. These results confirm the regulation of energy metabolism as one of SMI’s cardioprotective mechanisms. While according to GO enrichment analysis, among the DEPs in SMI: Mod group, the top three enriched BP (organelle organization, actin filament-based process and actin cytoskeleton organization), MF (cytoskeletal protein binding, actin binding, and actin filament binding), and CC (cytoskeletal part, cytoskeleton actin, and cytoskeleton) categories (Figure 5) indicate most of them belonging to actin cytoskeleton-related and actin-binding proteins. In addition, we can find some isotype actin proteins such as actin (ProteinID: P60711 and P63259), alpha-actinin-1 (ProteinID-Q9Z1P2), proto-oncogene tyrosine-protein kinase Src (ProteinID-Q9WUD9) and myosin regulatory light chain 12 B (ProteinID-A0A0G2JSW0) which were all involved in the top enriched KEGG pathways such as tight junction, regulation of actin cytoskeleton, leukocyte transendothelial migration, platelet activation, and oxytocin signaling pathway (Figure 6). Moreover, among these proteins, actin (ProteinID-P60711, GN = Actb), proto-oncogene tyrosine-protein kinase Src (ProteinID-Q9WUD9, GN = Src) and alpha-actinin-1 (ProteinID-Q9Z1P2, GN = Actn1) were also the top high-connectivity degree of proteins in PPI networks (Figure 7). Therefore, because of actin proteins’ important role in muscle contraction, we can speculate regulation of cardiac contractility might be involved in SMI’s cardioprotective effects.

The biological pathway analysis helps reveal what biological reactions that drug intervention can trigger, and it is most important for guidance of therapeutic target discovery and treatment strategy suggestion. Some pharmacological studies reported SMI’s effects of anti-inflammatory, anti-oxidation, and suppression of cell apoptosis through pathways such as caspase-3, mTOR, JNK, AKT, ERK1/2, and AMPK (Li et al., 2019; Zhu et al., 2019; Cao et al., 2020). Recently, Jiang et al. (Jiang et al., 2020) used systemic pharmacology and chemoinformatics to predict signaling pathways, biological processes, and targets of Shengmai Yin (including SMI) therapeutic role in coronary heart disease. Interestingly, in this serum proteome, we found 100 DEPs owing to SMI’s intervention could be mapped to 184 signaling pathways by KEGG database, and these pathways cover most of the reported. Moreover, from the top enriched KEGG pathways, the significant pathways such as tight junction, regulation of actin cytoskeleton, leukocyte transendothelial migration, oxytocin signaling pathway, focal adhesion, and vascular smooth muscle contraction are the first findings for SMI’s cardioprotective effects. While the regulation of biological processes involving these pathways actually plays key roles in cardiovascular pathophysiology. Tight junctions are intercellular adhesion complexes in epithelia and endothelia that control paracellular permeability (Zihni et al., 2016), and regulating it in human cardiac endothelial cells is reported to reduce doxorubicin-induced cardiotoxicity by reducing endothelial cell permeability (Wilkinson et al., 2018). Actin cytoskeleton, composed of actin polymers and associated actin-binding proteins, is responsible for mediating various important cellular processes such as cell structural support, axonal growth, cell migration, organelle transport, and phagocytosis (Lee and Dominguez, 2010). And at least the role and regulation of actin cytoskeleton are related to cardiac contractile function maybe through modulating smooth muscle cell migration (Tang and Gerlach, 2017) or myocardial Ca^2+^ overloaded (Miura et al., 2010). So combining with other top pathways of vascular smooth muscle contraction (Bai et al., 2020) and cGMP–PKG signaling pathway (Munzel et al., 2003) found in this study, we can speculate SMI’s effects on ISO-induced myocardial ischemia might partly regulate vessel and cardiac contractility. In addition, we found other important pathways related to inflammation induction in cardiovascular pathology such as leukocyte transendothelial migration, platelet activation, and MAPK signaling pathway-fly (Muller, 2011; Yuan et al., 2017; Papaconstantinou, 2019), which add on the evidence of SMI’s anti-inflammatory effect. The regulation of the oxytocin signaling pathway and focal adhesion which may partly contribute to SMI’s prevention of cell apoptosis because oxytocin was reported to protect the heart and blood vessels from immunometabolic injuries and the resultant inflammation and apoptosis (Wang et al., 2019), and focal adhesion kinase (FAK) mediates adenosine and homocysteine-induced endothelial cell apoptosis (Lu and Rounds, 2012). Taken together, this global serum proteomic profile highlights the multiple pathways of SMI’s cardioprotective effects, except for regulation of the energy metabolism, which might at least include reduction in endothelial cell permeability, regulation of vessel and cardiac contractility, anti-inflammation, and prevention of cell apoptosis. However, their clear mechanism needs further confirmation and more research.

TCM formula injection has potential on clinical uses, but its safety deserves special attention. *In vivo* process studies including pharmacokinetics, pharmacodynamics, and their combined studies are of significance for TCM formula’s effectiveness and safety because they help guide the reasonable clinical uses. At present, pharmacokinetic studies of the TCM formula have gained great progress because of the advancement of analytical technology (Crotti et al., 2017). However, pharmacodynamic studies still face much challenge such as lack of precise and accessible effect indices. Serum proteomics not only reveals targets and mechanism in large scale but also helps screen potential biomarkers which would provide candidate serum effect indices benefit for TCM’s pharmacokinetics–pharmacodynamics studies. In this proteomics, we noticed 10 common DEPs in Mod: Con and SMI: Mod groups (Table 3) according to the Venn diagram analysis, and six of them, carboxylic ester hydrolase (ProteinID-D4AA05), UMP-CMP kinase (ProteinID-Q4KM73), uncharacterized protein (ProteinID-M0R5J4), glyceraldehyde-3-phosphate dehydrogenase (ProteinID-M0R590), Fas apoptotic inhibitory molecule 3 (ProteinID-Q5M871), and RCG21066 (ProteinID-D3ZJW6), were significantly up- or downregulated in serum of ISO-induced myocardial ischemia rats while were significantly back-regulated in serum of SMI pretreatment rats. These six proteins should become potential biomarkers for SMI’s cardioprotective effects after their biological function interpretation. We verified three of them, glyceraldehyde-3-phosphate dehydrogenase (GAPDH), Fas apoptotic inhibitory molecule 3 (FAIM3), and uncharacterized protein, by PRM analysis which supports the results of their regulation change among groups from TMT proteomics data. Our study found SMI attenuated the upregulation of serum GAPDH and M0R5J4 (uncharacterized protein) in ISO-induced myocardial ischemia rats, which should reflect SMI’s overall regulation on these biomarkers. GAPDH is a multifunctional protein with multiple cytoplasmic, membrane, and nuclear functions, except for its metabolic function as a classical glycolytic enzyme in cellular energy production (White and Garcin, 2017). It is notable that GAPDH is recently implicated in initiation of apoptosis including promotion of cardiomyocyte apoptosis (You et al., 2013), but on the other hand, its downregulation would result in cellular ATP depletion during smooth muscle cell energy metabolism (Sukhanov et al., 2006) and starvation-induced cardiomyocyte apoptosis (Yao et al., 2012). Thus, it can conclude the complex alteration of GAPDH in cardiovascular pathophysiology, but these changes would finally comprehensively reflected in serum as its level of upregulation, and it has also been early reported that the elevated serum GAPDH activity usually appears to be a potentially adjunct in the early detection of acute myocardial infarction (Karliner et al., 1971). Therefore, based on the multiple pathways of SMI’s cardioprotective effects, we speculate SMI can synthetically attenuate abnormal upregulation of serum GAPDH, and the underlying mechanism might at least include GAPDH-related improvement of energy metabolism and prevention of cell apoptosis. The Fas apoptotic pathway is one of the mechanisms in cardiomyocyte apoptosis which plays an important role in ischemic heart disease (Lee et al., 2003). FAIM3, belongs to the family of Fas apoptotic inhibitory molecule, is a less well-known gene which may play a role in the immune system processes, and it may protect cells from Fas-, TNF-alpha-, and FADD-induced apoptosis (Hitoshi et al., 1998). In this study, we first found the abnormal downregulation of FAIM3 in serum proteome from ISO-induced myocardial ischemia rats, and SMI significantly upregulated it, which implies FAIM3 as a sensitive biomarker for directing SMI’s anti-apoptosis effect, although its associated mechanism needs to be further confirmed. Further consideration from this study, we found some serum biomarkers which can potentially indicate SMI’s effect on ISO-induced myocardial ischemia in rats. However, their clinical significance of SMI treating cardiovascular diseases deserves additional evaluation based on clinical cases.

## Conclusion

In conclusion, we performed serum proteomic analysis on SMI’s cardioprotective effects on ISO-induced myocardial ischemia injury in rats using the TMT quantitative proteomic technology in this study. 227, 100, and 228 serum DEPs in model + control, SMI + model, and SMI + control group were identified, respectively, which are much more than what we found in previous myocardial tissue proteomics. After bioinformatics analysis, we can speculate the comprehensive mechanism of SMI’s effect on ISO-induced myocardial ischemia injury in rats, which might include regulation of energy metabolism, reduction of endothelial cell permeability, regulation of vessel and cardiac contractility, anti-inflammation, and prevention of cell apoptosis. Furthermore, based on the common DEPs which were regulated in ISO-induced myocardial ischemia rats and back-regulated in SMI pretreatment myocardial ischemia rats, we identified three serum biomarkers of glyceraldehyde-3-phosphate dehydrogenase (GAPDH), Fas apoptotic inhibitory molecule 3 (FAIM3), and uncharacterized protein (M0R5J4) verified by the PRM analysis. SMI can alleviate the abnormal elevation of serum GAPDH through synthetic regulation and upregulate serum FAIM3 for its anti-apoptosis effect. These proteins might be potential serum biomarkers for directing SMI’s cardioprotective effects. This is the first serum proteomics study of TCM’s effects on ischemic heart disease using SMI as a representative, and it helps reveal the comprehensive mechanism and identify serum biomarkers of SMI’s cardioprotective effects from a global perspective. However, its explicit mechanism needs to be further confirmed, and the relationship of SMI’s comprehensive mechanism with its nature of multicomponent and multi-target deserves the following exploration.

## Data Availability

The original contributions presented in the study are included in the article/Supplementary Material, further inquiries can be directed to the corresponding author.
