# Supplementary figures and images for: A Quantitative Serum Proteomic Analysis Helps to Explore the Comprehensive Mechanism and Identify Serum Biomarkers of Shengmai Injection’s Effect on Isoproterenol-Induced Myocardial Ischemia in Rats
Source: Front Pharmacol. 2021 Apr 28;12:666429. doi: 10.3389/fphar.2021.666429 (PMC8113823; doi:10.3389/fphar.2021.666429)

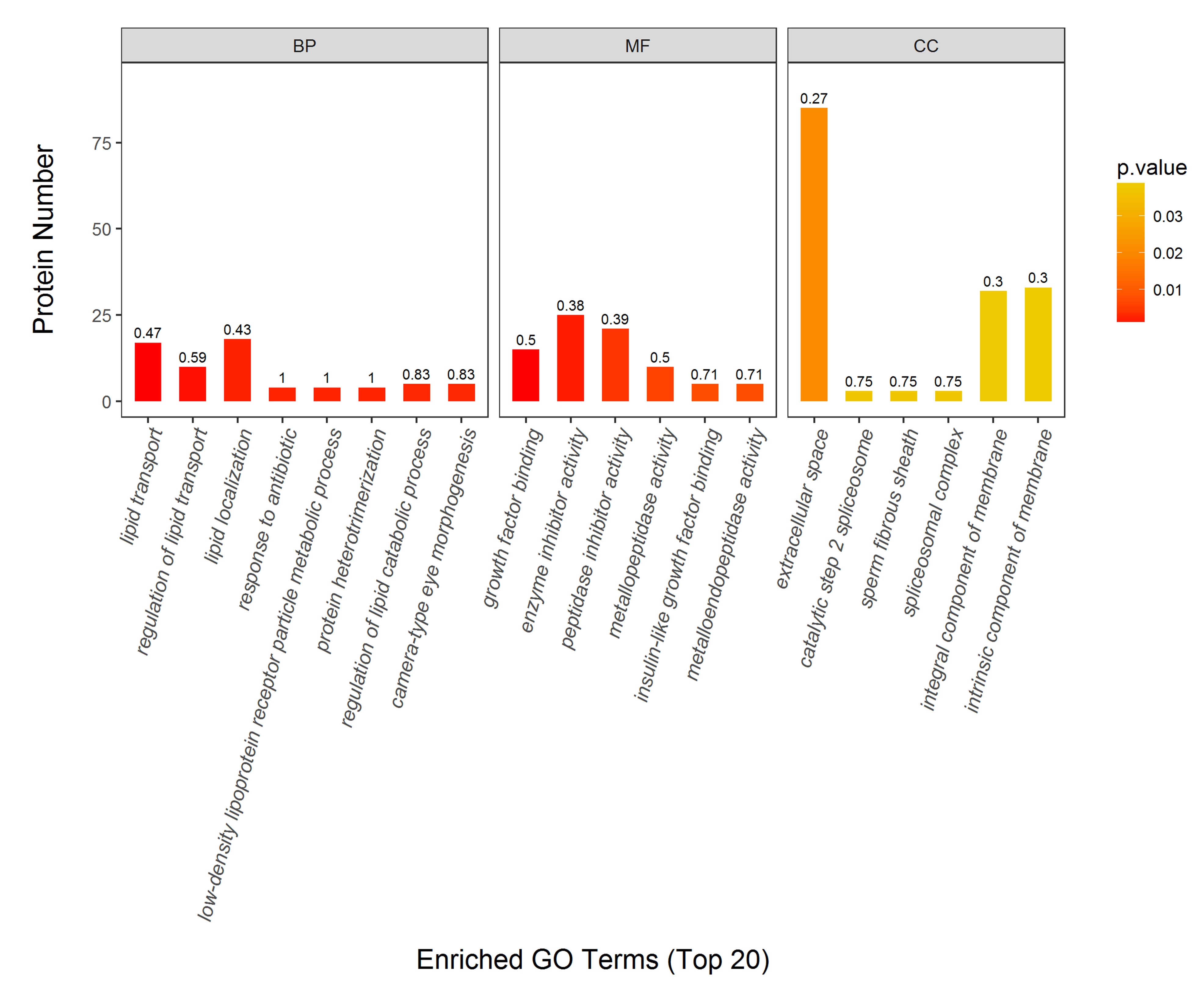

Supplement: Supplementary file 1 [file image3.tif]

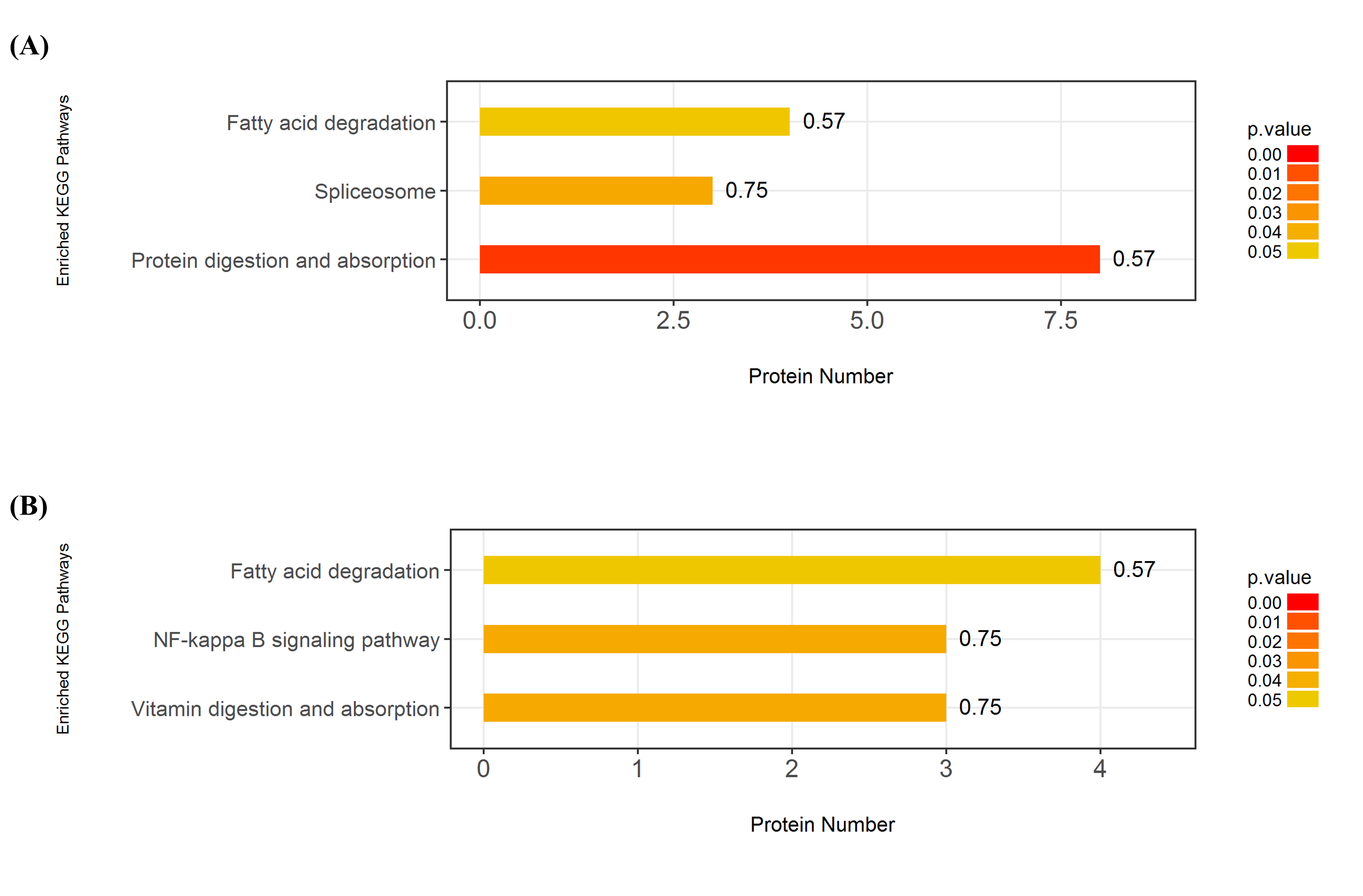

Supplement: Supplementary file 2 [file image4.tif]

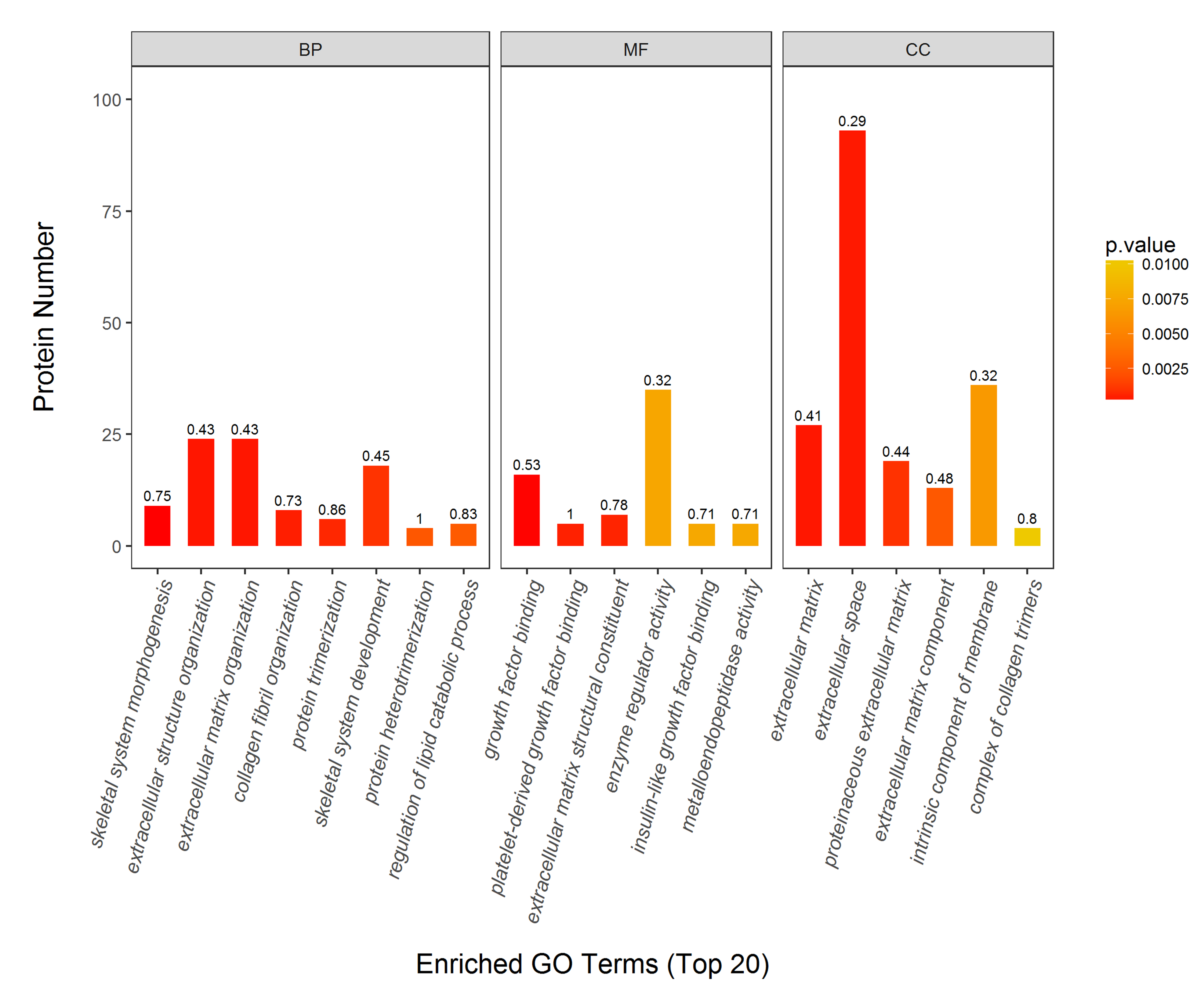

Supplement: Supplementary file 3 [file image2.tif]

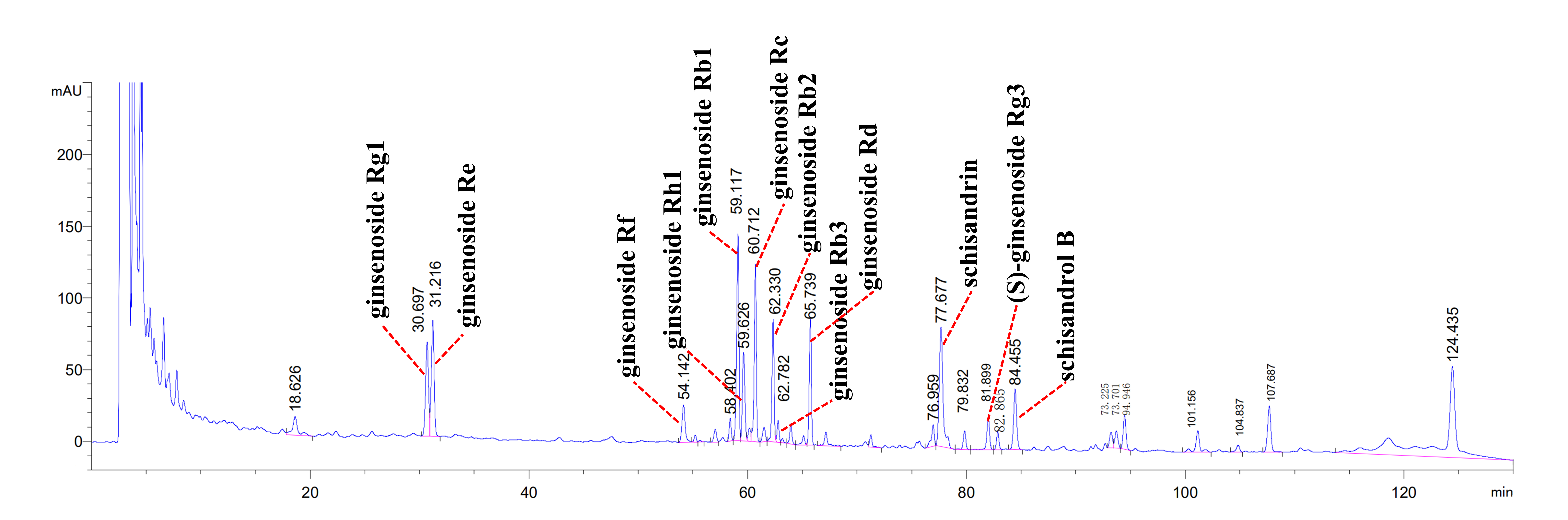

Supplement: Supplementary file 4 [file image1.tif]

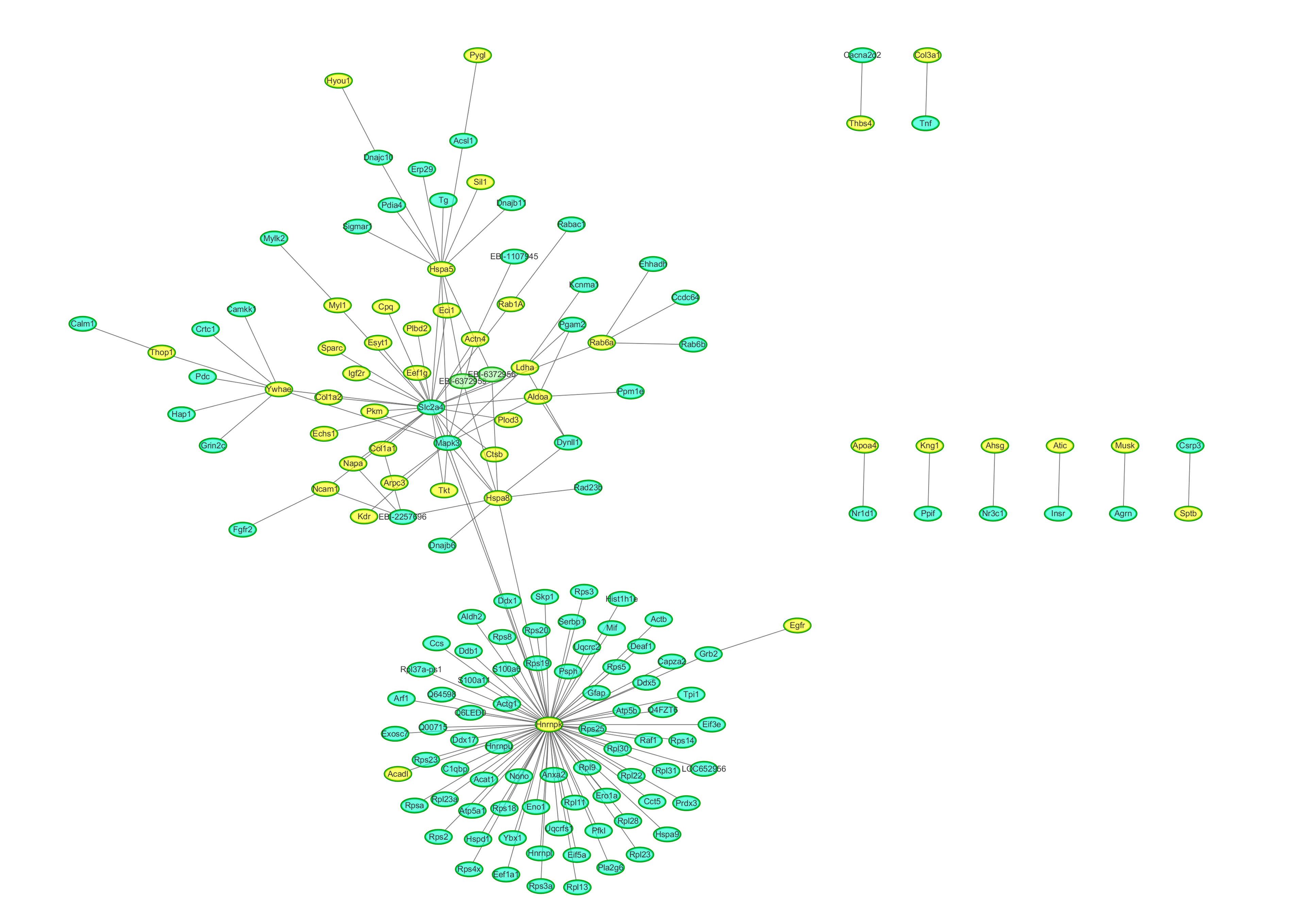

Supplement: Supplementary file 5 [file image5.tif]
